# REASSESSMENT OF FATTY INFILTRATION BY MAGNETIC RESONANCE TEN YEARS AFTER ROTATOR CUFF INJURY REPAIR

**DOI:** 10.1590/1413-785220233103e262497

**Published:** 2023-07-17

**Authors:** ALBERTO NAOKI MIYAZAKI, LUCIANA ANDRADE DA SILVA, CAIO SANTOS CHECCHIA, JULIO CESAR DO AMARAL MUSSATTO, VINICIUS MARQUES DE MORAES, GUILHERME DO VAL SELLA

**Affiliations:** 1 Santa Casa de Misericordia de Sao Paulo, Faculdade de Ciencias Medicas, Departamento de Ortopedia e Traumatologia, Grupo de Cirurgia do Ombro e Cotovelo, São Paulo, SP, Brazil.

**Keywords:** Rotator Cuff, Muscular Atrophy, Magnetic Resonance Spectroscopy, Manguito Rotador, Atrofia Muscular, Espectroscopia de Ressonância Magnética

## Abstract

Quantitative assessments of rotator cuff muscle changes after successful tendon repair are scarce. On the other hand, semiquantitative and subjective assessments are more abundant, but their findings are controversial. One hypothesis about this divergence is that there is an immediate decrease in the proportion of fatty infiltration after surgical repair.

**Objective:**

Reassess fatty infiltration and muscle trophism of the rotator cuff after ten years of repair.

**Methods:**

Prospective comparison study. A total of 10 patients diagnosed with rotator cuff injury underwent repair of the lesion, and MRI of the affected shoulder was performed in the preoperative, immediate postoperative, and late postoperative periods (ten years). A comparative study was performed at every moment.

**Results:**

At 5% significance level, the mean of the immediate postoperative period was higher for the variable trophism and true muscle percentage. Fatty infiltration showed no difference in the three periods observed.

**Conclusion:**

Fatty infiltration does not change in the three periods evaluated and muscle trophism is greater in the immediate postoperative period. After ten years of rotator cuff repair, muscle trophism and fatty infiltration remain with statistically significantly equal results when compared to the preoperative period. ***Level of Evidence II, Prospective Comparison Study.***

## RESUMO

Avaliações quantitativas das mudanças musculares do manguito rotador após reparos bem-sucedidos são escassas. Em contrapartida, avaliações semiquantitativas e subjetivas são mais abundantes, porém com achados controversos. Uma hipótese sobre essa discrepância é que a diminuição imediata na proporção de gordura que ocorre logo após o reparo.

**Objetivo:**

Reavaliar a infiltração gordurosa e o trofismo muscular do manguito rotador passados dez anos do reparo.

**Métodos:**

Estudo prospectivo comparativo realizado com dez pacientes diagnosticados com lesão do manguito rotador que foram submetidos a reparo da lesão e exames de ressonância magnética do ombro acometido no pré-operatório, no pós-operatório imediato e no pós-operatório tardio (dez anos), a fim de comparar as mudanças musculares em cada momento.

**Resultados:**

Ao nível de significância de 5%, a média do pós-operatório imediato foi superior para as variáveis trofismo e porcentagem muscular verdadeira. A infiltração gordurosa não apresentou diferença nos três períodos observados.

**Conclusão:**

A infiltração gordurosa não se altera nos três períodos avaliados, e o trofismo muscular é maior no pós-operatório imediato. Após dez anos do reparo do manguito rotador, o trofismo muscular e a infiltração gordurosa se mantêm com resultados estatísticos significativamente iguais quando comparados com o pré-operatório. **Nível de Evidência II, Estudo Prospectivo Comparativo.**

## INTRODUCTION

Rotator cuff injuries are common causes of musculoskeletal problems with a prevalence of 15 to 51% in the population, with a higher incidence in patients over 50 years.^1^ After rotator cuff injury, muscle retraction occurs, causing degenerative tissue changes (fatty infiltration and atrophy), ^(^ ^2^ which some authors consider progressive and irreversible. ^(^ ^3^ ^)-(^ ^5^ Both muscle atrophy and fatty infiltration have a substantial influence on the clinical results of rotator cuff repair and on the re-rotation rate, and therefore interfere with management. ^(^ ^5^ ^),(^ ^6^

Quantitative assessments of rotator cuff muscle changes after successful tendon repair are scarce. ^(^ ^7^ In contrast, semiquantitative and subjective assessments are more abundant, but their findings are controversial: some authors report that the degenerative changes are irreversible^5^ ^),(^ ^8^ ^),(^ ^9^ whereas others report recovery of muscle atrophy or even fatty infiltration. ^(^ ^10^ ^)-(^ ^12^ One hypothesis about this divergence is that there is an immediate decrease in the proportion of fat (in addition to an increase in the occupancy of the muscle in its fossa) soon after surgical repair. This is probably due to the traction exerted on the tendon at the time of its repair^6^ ^),(^ ^13^ since the muscle-tendon unit is pulled in the coronal plane of the scapula and the evaluation is usually performed in a fixed sagittal plane perpendicular to that of the traction. ^(^ ^14^

Precisely to study this hypothesis, in a previous study^6^ we evaluated degenerative changes in the muscles of the supraspinatus comparing preoperative and immediate postoperative (POi) magnetic resonance imaging (MRI) scans of ten patients subjected to repair of rotator cuff injury, and concluded that there is an immediate increase in the muscle occupancy of the supraspinatus in its fossa.

This study aims to reevaluate the same patients from the first study, approximately ten years later, and to compare the current results with the previous ones. ^(^ ^6^ Our hypotheses are that over the course of the follow-up: (1) in cases with maintenance of repair integrity, fatty infiltration is unchanged and muscle trophism is increased; (2) in cases of loss of repair, fatty infiltration is increased and muscle trophism is decreased.

## METHODS

This study was conducted from September 2011 to December 2011 in a tertiary hospital located in the city of São Paulo. A total of 10 patients (four men and six women) diagnosed with complete rotator cuff injury (MRL) were subjected to repair of the lesion, arthroscopic or open. MRI examinations of the affected shoulder were performed in the preoperative (at most one month before surgery) (Pre-op) and immediate postoperative (IPO) (at most one month after surgery). On that occasion, the area and muscle volume in the supraspinatus fossa were evaluated, comparing the pre- and immediate postoperative measurements. For this study, a new MRI was performed in a third moment, about ten years after surgery (POlate) (mean of 9.6 years, ranging from 9.5 to 10.1 years).

The inclusion criteria in this study were the same: patients previously subjected to rotator cuff injury repair and evaluated in the previous study^6^ with sufficient data for comparison of the preoperative, immediate postoperative, and ten years postoperative. A total of two of these 10 initial cases were excluded due to the impossibility of contact for reevaluation, plus one case that underwent a reverse total shoulder arthroplasty and another that refused to be part of the study. Thus, six patients were reassessed.

The six patients had a mean age of 71.6 years at the time of the last reassessment (65 to 75 years). They were five women and one man. In all cases, the lesions affected exclusively the supraspinatus and infraspinatus tendons. Table 1 shows other data regarding the types of lesions found and the procedures performed.

**Table 1:** Data on patient injury and surgical procedure.

	Size of injury*	Surgical procedure	Acromioplasty	Tenotomy LHB
1	Medium	Arthroscopy	Yes	No
2	Large	Open	Yes	No
3	Medium	Arthroscopy	Yes	Yes
4	Medium	Arthroscopy	Yes	No
5	Large	Arthroscopy	No	Yes
6	Large	Arthroscopy	Yes	No

LHB: long head of the biceps brachii muscle.

*According to Cofield’s classification. ^(^ ^15^

All MRI scans were performed in a high-field 1.5 Tesla equipment (Achieva, Philips Medical Systems, Holanda B.V.^®^). T1- and T2-weighted sagittal and coronal oblique images were obtained with reference to the glenoid cavity, as well as proton density-weighted images in the axial plane.

The MRI images were analyzed in the report program of the Department of Radiology and Diagnostic Imaging (IMPAX Agility 8.1.2 SP7.7 - Agfa Healthcare^®^) and evaluated by two radiologists. One of them was in the specialization phase in the area of musculoskeletal radiology and the other was already sub-specialized in the area for more than 15 years. Both evaluators had access to the patients’ previous images and knew that the estimation was intended for a scientific study. There was no blinding. Each one estimated the measures only once, and the result used in this study was the average of these two measures for each variable.

**Figure 1:**
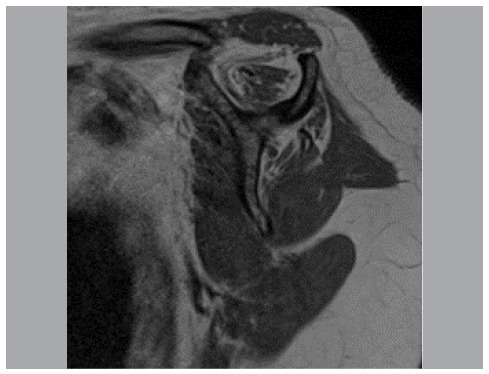
Sagittal section used to assess T1-weighted fatty infiltration.

The T1-weighted oblique sagittal sequence, in which fat is observed with high signal and muscle with intermediate signal, was the sequence chosen for the evaluations. The chosen section was the most lateral in which the spine of the scapula is evidenced in continuity with the body, forming a “Y” (Figure 1). Once selected, the image was saved as a file and opened in Adobe Photoshop CS6^®^. The quick selection tool, which automatically selects areas of similar brightness, was used.

**Figure 2:**
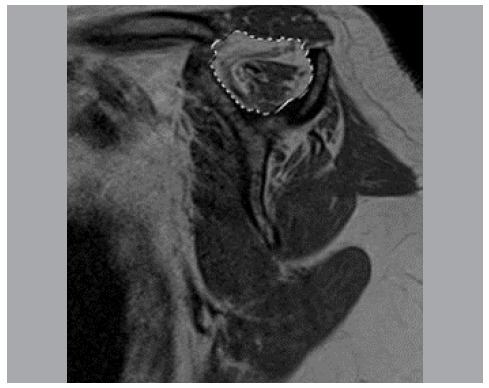
Area of the supraspinatus fossa.

**Figure 3:**
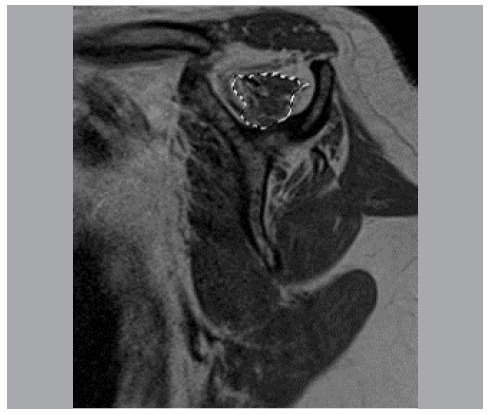
Area of the supraspinatus muscular belly.

**Figure 4:**
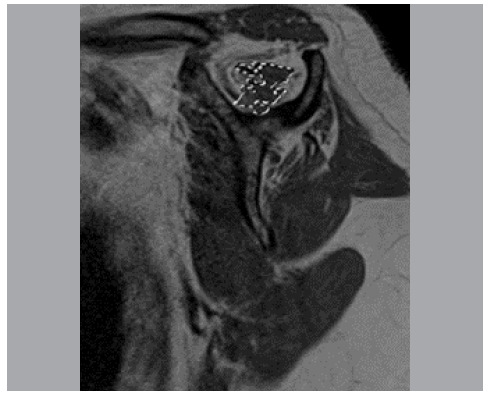
Area of remaining supraspinatus muscle fibers.

For each MRI in the entire study, three measurements were performed: area of the supraspinal fossa (called “Fossa”) (Figure 2); area of the supraspinatus muscular belly (contour of muscle mass) (called “Belly”) (Figure 3); and area of remaining supraspinatus muscle fibers (contour of high-signal regions on T1-weighted sequences) (called “musc”) (Figure 4).

From these three measurements, two other proportions were estimated: Muscle Trophism (= Belly/Fossa) and Fatty Infiltration (= 1-[Musc/Belly]). The variation between the proportions of POlate and IPO was interpreted as a dependent variable.

The Friedman test was used to evaluate if muscle trophism, fatty infiltration, and muscle percentage changed over time, that is, if time (preoperative, postoperative, and evaluation after ten years) influenced the variables mentioned above.

This work was submitted and approved by the Research Ethics Committee under opinion: CAAE 45571621.7.0000.5479. All patients in the study signed the informed consent form.

## RESULTS

Table 2 presents the muscle percentages of the supraspinatus muscle in the suprascapular fossa and the fatty infiltration of the six patients on three occasions: preoperative, immediate postoperative, and late postoperative.

**Table 2:** Muscle percentages of the supraspinatus muscle in the suprascapular fossa.

	Preoperative		Postoperative		Postoperative ten years later	
	SF	FI	SF	FI	SF	FI
1	0.323	0.94	0.355	0.84	0.162	0.84
2	0.397	0.89	0.497	0.89	0.376	0.93
3	0.269	0.72	0.319	0.79	0.253	0.79
4	0.419	0.84	0.461	0.75	0.325	0.81
5	0.173	0.78	0.226	0.75	0.218	0.85
6	0.313	0.78	0.484	0.97	0.385	0.78

SF: area of muscle occupancy in the supraspinatus fossa; FI: fatty infiltration (%muscle).

The preoperative group had a mean value of 0.315 for the true muscle percentage in the fossa, ranging from 0.173 to 0.419; the immediate postoperative group had a mean value of 0.389, ranging from 0.226 to 0.497; and the late postoperative group had a mean value of 0.286, ranging from 0.162 to 0.385.

### Muscle trophism

Friedman’s test rejected the hypothesis that conditions did not influence trophism (p = 0.009).

**Figure 5:**
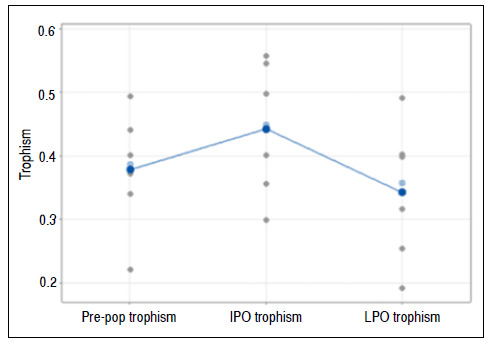
Graph of individual values for the trophism variable, emphasizing the means and medians. Pre-op: preoperative; IPO: immediate postoperative; LPO: late postoperative.

At the 5% significance level, the mean of the variable Trophism was higher in the immediate postoperative compared with the other conditions (Figure 5).

### Fatty infiltration

Friedman’s test did not rejected the hypothesis that conditions did not influence trophism (p = 0.638).

**Figure 6:**
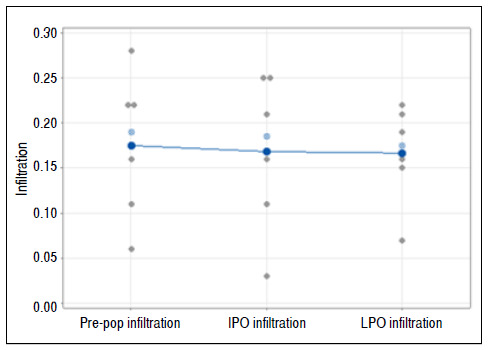
Graph of individual values for the infiltration variable, emphasizing the means and medians. Pre-op: preoperative; IPO: immediate postoperative; LPO: late postoperative.

We thus conclude that there was no difference in the infiltration variable in the three occasions observed (Figure 6).

### Muscle percentage

Friedman’s test rejected the hypothesis that the conditions did not influence Musc (p = 0.009)

**Figure 7:**
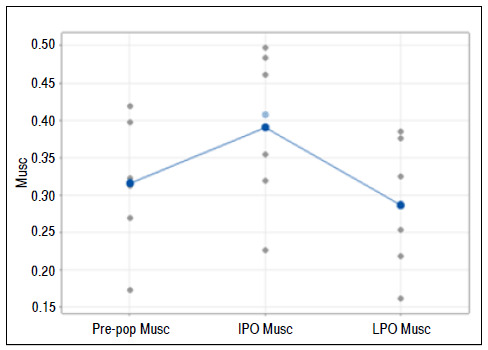
Graph of individual values for the Musc variable, emphasizing the means and medians. Pre-op: preoperative; IPO: immediate postoperative; LPO: late postoperative; Musc: area of remaining supraspinatus muscle fibers.

At the 5% significance level, the mean of the variable Musc was higher in the immediate postoperative compared with the other conditions (Figure 7).

Next, the Anderson-Darling goodness of fit tests were performed for all variables, concluding that the distributions of all variables fitted the normal distribution. Thus, to evaluate the existence of linear relationships, Pearson’s correlation coefficient was used.

At 5% significance level, only IPO Trophism and Pre-op Trophism showed a linear (increasing) relationship (p = 0.007).

At 5% significance level, no linear correlation was significant in relation to fatty infiltration.

At 5% significance level, IPO Musc and Pre-op Musc (p = 0.023) and IPO Musc and LTO Musc (p = 0.050) showed linear (increasing) relationship.

The small sample size made it impossible to analyze the impact of the variable “reinjury” on the outcomes “Trophism,” “Fatty infiltration,” and “% Musc”. Therefore, it was presented only descriptively (Table 3).

**Table 3:** Descriptive statistics for the variables trophism, infiltration, and muscle, in the three conditions, in the groups without reinjury and with reinjury.

Variable	Pre-op trophism		IPO trophism		LPO trophism		Pre-op infiltration		IPO infiltration		LPO infiltration		Pre-op Musc		IPO Musc		LPO Musc	
**Reinjury**	No	Yes	No	Yes	No	Yes	No	Yes	No	Yes	No	Yes	No	Yes	No	Yes	No	Yes
**Medium**	0.421	0.357	0.5275	0.4005	0.4465	0.2903	0.165	0.18	0.07	0.2175	0.145	0.1775	0.355	0.296	0.4905	0.3402	0.3805	0.2395

Pre-op: preoperative; IPO: immediate postoperative; LPO: late postoperative; Musc: area of remaining supraspinatus muscle fibers.

## DISCUSSION

Muscle atrophy and fatty infiltration lead to loss of muscle elasticity and strength and are associated with outcomes of surgical rotator cuff repair. ^(^ ^13^ Both atrophy and fatty degeneration progress with non-surgical treatments. ^(^ ^16^ Repair results in decreased pain and functional improvement of the shoulder, but the effects of repair on muscle trophism and fatty infiltration are still controversial. ^(^ ^17^

In 2007, Liem et al. ^(^ ^5^ conducted a study with 53 patients subjected to repair of rotator cuff injuries (RCI), evaluated with MRI after two years, showing an irreversibility of both trophism and fatty infiltration. These results were confirmed by Gladstone et al. ^(^ ^8^ in the same year. In the study, the 38 patients subjected to repair and evaluated one year later with MRI showed irreversibility of trophism or fatty infiltration, even in successful repairs.

In 1997, Thomazeau et al., ^(^ ^18^ conducted a study with 30 patients subjected to RCI repair and reevaluated two years later: there was reversibility of muscle trophism in half of the patients in whom the repair remained intact. Gerber et al. ^(^ ^19^ did not obtain the same result regarding trophism with patients subjected to repair of extensive lesions and evaluated two years later, but did show a significant improvement in fatty infiltration. 

In 2016, Parker et al. ^(^ ^13^ evaluated 47 patients with MRI studies, finding a small statistical improvement in trophism after 2 years of follow-up (11.3% to 13.9 %), and it was not possible to prove a statistical improvement in relation to fatty infiltration.

In this controversial scenario, Kim, Yoo and Jeong^17^ conducted a study with comparative times points (preoperative; immediate postoperative [IPO] and late postoperative [LPO] at six months) to further elucidate this issue. The authors showed significant improvements in both muscle trophism and fatty infiltration in the IPO compared to the preoperative. These results are partially in line with our study, in which muscle tropism was significantly improved in IPO, but fatty infiltration was not. Kim, Yoo and Jeong^17^ also compared preoperative with LPO, as well as IPO with LPO. Their results showed no significant improvement in fatty infiltration or muscle trophism comparing either the preoperative with LPO or IPO with LPO.

Our study performed a prolonged follow-up (with a LPO close to ten years) at three time points (preoperative, IPO, and LPO). It evidenced that fatty infiltration does not change at any time point and that muscle trophism and the true muscular percentage of supraspinatus are higher at IPO compared to both preoperative and LPO. It also showed no difference for any of the variables analyzed in the preoperative and the LPO.

We think that the improvement in muscle trophism in the immediate postoperative can be explained in two ways: either there is a true improvement, or there is a misinterpretation. The reduction of the tendon in the surgical procedure changes the muscle area evaluated, while the standardized scapular plane is maintained, generating a bias. ^(^ ^13^ Another factor that alters the interpretation of the exam in cases of IPO evaluation is that the presence of saline solution in the subacromial space or local edema may hinder the evaluation. ^(^ ^17^

Over time, RCI in cases not treated surgically lead to the increase of muscle trophism and fatty infiltration, ^(^ ^16^ but the outcomes of cases with surgical repair and reinjury may differ; we thus sought to evaluate, with statistical significance, the relationship of repair integrity and the result of the variables trophism and fatty infiltration in LPO. However, due to the low number of patients reevaluated, it was not possible. We believe that further studies with larger samples are important.

The strengths of this study were: (1) imaging studies performed with MRI; (2) standardization in the performance of the exams (3) long postoperative follow-up (approximately ten years). The limitations were: (1) small sample size (six patients) (2) lack of correlation between imaging results and functional results.

## CONCLUSION

Muscle trophism and the true muscle percentage of the supraspinatus in its fossa are higher in the immediate postoperative compared to the preoperative and late postoperative (ten years). Fatty infiltration does not change.

After ten years of rotator cuff repair, muscle trophism and fatty infiltration remain statistically significantly equal when compared with the preoperative moment.
